# Shyness Weakens the Agreeableness-Prosociality Association via Social Self-Efficacy: A Moderated-Mediation Study of Chinese Undergraduates

**DOI:** 10.3389/fpsyg.2019.01084

**Published:** 2019-05-14

**Authors:** Peng Sun, Zhen Liu, Qingke Guo, Junyi Fan

**Affiliations:** ^1^School of Psychology, Shandong Normal University, Jinan, China; ^2^Beijing Key Lab of Applied Experimental Psychology, Faculty of Psychology, Beijing Normal University, Beijing, China; ^3^Guangxi University and College Key Laboratory of Cognitive Neuroscience and Applied Psychology, Guangxi Normal University, Guilin, China

**Keywords:** agreeableness, prosocial behavior, shyness, social self-efficacy, moderated mediation model

## Abstract

Using a sample of 1383 undergraduate students (*M*_age_ = 20.06, *N*_female_ = 817), this study tested a moderated mediation model in which shyness moderated the association between agreeableness and prosocial behavior, as well as the relation between agreeableness and social self-efficacy (SSE). Results showed (when gender, age, and family socio-economic status were controlled) that agreeableness exerted a positive effect on prosocial behavior (PSB) toward three types of recipients (i.e., family members, friends/acquaintances, strangers), and this effect was mediated by SSE and moderated by shyness. The relationships between agreeableness and PSB were more positive under low shyness than that under high shyness condition. In addition, shyness also moderated the first stage of mediation model (i.e., the agreeableness-SSE association), showing that the relation between agreeableness and SSE was more positive under low shyness than that under high shyness condition. Identifying the moderation effect of shyness provides evidence that personality traits may operate in an interactive manner. This may shed new light on why there are inconsistent findings regarding the agreeableness-prosociality association.

## Introduction

An increasing interest has been focused on individual differences in prosociality (e.g., Penner et al., 2005). Prosocial behavior (PSB), the tendency to help, to donate, to comfort, and to care, can be partly attributed to differences in particular personality traits (e.g., Caprara et al., 2012; Oda et al., 2014; Habashi et al., 2016). Recently, agreeableness of the “Big Five” personality model (McCrae and Costa, 2010), has been regarded as the most important dimension influencing PSB (Graziano et al., 2007; Habashi et al., 2016). For example, agreeableness has been found to be associated with both prosocial emotions (i.e., empathic concern and personal distress) and helping behaviors (Habashi et al., 2016).

However, the association between agreeableness and PSB may be also affected by situational and other dispositional factors. Habashi et al. (2016) proposed that agreeableness may play a more important role in influencing helping behaviors in situations where empathic concern for the victim is not strongly elicited. But when empathic concern is explicitly induced, the difference in prosociality between high and low agreeable people become greatly reduced. In the above citation, when required to put themselves in the situation of the victim, the participants, regardless of their level of “prosocial” personality, are more prone to prosocial engagement (Habashi et al., 2016). Oda et al. (2014) investigated the relationship between personality and altruistic behaviors in daily life. They found that agreeableness was positively associated with altruism toward friends/acquaintances, but was not associated with altruism toward family members. This suggests that kin altruism may be hard wired and strong, and therefore cannot be accounted for by individual differences in personality (Ben-Ner and Kramer, 2011). In addition, a recent study exploring determinants of individuals’ charitable behavior (donations of time and money) found that agreeableness was only positively and significantly related with monetary donations, but not with time donations (Brown and Taylor, 2015).

It has been suggested that agreeableness alone cannot be responsible for all situational variability in PSB, especially when specific abilities are required. Whether a prosocial disposition can translate into actual behavior may depend on how one perceives oneself in social settings (Caprara et al., 2012). PSB occurring in social settings requires different social competence and motivation to participate in various situations. For example, it is obvious that PSB toward non-kin, strangers, and time donations (e.g., volunteering) need higher social participation than PSB toward family members and friends, and monetary donations. Agreeableness may facilitate people to endorse prosocial values and show prosocial tendencies, but such values or tendencies may not always turn into actual PSB in daily life, especially when high social participation is required (Caprara et al., 2012).

Shyness may be such a factor that interacts with agreeableness in influencing PSB. Shyness is characterized by behavioral inhibition and avoidance in social interactions (Zimbardo, 1977; Hammick and Lee, 2014). Shyness is closely associated with self-consciousness, lack of self-confidence, social anxiety, and excessive concern of negative evaluations (Briggs, 1988). Existence of these features may significantly weaken agreeable individuals’ motivation to act prosocially. Thus, the present study aims to investigate the association between agreeableness and PSB from social-cognitive perspective which may provide a more unified view of personality process (Hampson, 2012) in order to understand the agreeableness-prosociality association more clearly. Specifically, this study was intended to investigate the moderating role of shyness and the mediating role of social self-efficacy (SSE) in the relation of agreeableness and PSB.

### Agreeableness and Prosocial Behavior

Agreeableness is suggested to be the core component of prosocial personality (Ashton et al., 1998; Habashi et al., 2016). Existing literature mostly suggests that agreeableness is positively associated with prosocial emotions and behavior (Graziano et al., 2007). For example, agreeable individuals are more likely to engage in volunteering (Carlo et al., 2005) and money donation (Paunonen and Ashton, 2001; Yarkoni et al., 2015). Ashton et al. (1998) found that agreeable individuals allocated more money to a close friend and even a consistently uncooperative person. Consistently, a recent study found that agreeableness was uniquely and broadly associated with the amount of wealth allocated to the partners in various economic games (Zhao et al., 2016). But there are also some inconsistent findings. For example, agreeableness showed no or even negative relationship with prosocial orientation and giving in the dictator game (Ben-Ner and Kramer, 2011; Brocklebank et al., 2011), cooperative decision making in the prisoner’s dilemma (Hirsh and Peterson, 2009; Lönnqvist et al., 2011), and altruistic transfers in the public goods game (Kurzban and Houser, 2001). Inconsistent findings mentioned above suggest that there are other factors influencing the agreeableness-prosociality association.

### Shyness as a Moderator

A considerable number of people may label themselves as shy (Henderson et al., 2014). Identifying the role of shyness in influencing social functioning is important theoretically and practically. Shyness deserves more academic concern in Asian cultures, especially in China (e.g., Chen and French, 2008). Shyness is featured by insufficient self-confidence, lack of social skills, social anxiety, and behavioral inhibition in social settings (Zimbardo, 1977; Hammick and Lee, 2014). Shyness is often associated with less peer interactions and poor social adjustment (Coplan et al., 2004). As a construct, shyness is different from the well-known personality dimensions of introversion and neuroticism, serving as a bridge between the two (Briggs, 1988). Specifically, shyness is not a sub-factor of neuroticism or introversion alone, it is a primary factor contributing to both neuroticism and introversion. In addition, although shyness and agreeableness are interrelated, they are actually distinct personality traits (Cheek and Buss, 1981; Briggs, 1988). Agreeable people tend to be sociable, but low sociability (e.g., low friendliness and unwillingness to engage in social interactions) is not equivalent to shyness (e.g., feelings of awkwardness or apprehension in the presence of others). Existing literature further suggests that these two traits may interact to influence social behavior (Cheek and Buss, 1981; Santesso et al., 2004). That is, they may operate in an interactive manner rather in an additive manner.

Shy individuals are socially less competent and show less social skills in social settings (Rubin et al., 2009). They rarely initiate interpersonal interactions, and tend to mask their emotions (Eisenberg et al., 1995b). These features suggest that shyness is associated with less social engagement. PSB usually involves face-to-face interpersonal interactions and communications (Guo et al., 2018a). Therefore we propose that shyness may weaken the positive effect of agreeableness on PSB. In other words, though agreeableness contributes to prosocial values and tendencies, shyness may hinder such values or tendencies from translating into actual behaviors. So we propose that shyness can moderate the positive effect of agreeableness on prosociality.

### Social Self-Efficacy as a Mediator

Social self-efficacy refers to beliefs in one’s capacity to effectively handle interpersonal interactions, successfully initiate social contact, and maintain and develop friendships (Connolly, 1989; Bandura, 1997). People may have less incentive to undertake an activity unless they believe they are able to do so and can attain desired results from their actions (Bandura, 1997). Therefore, it is unlikely that people with insufficient SSE engage in prosocial actions that requires active interpersonal interactions. Hermann and Betz (2004) proposed that higher level of SSE may help people alleviate anxiety, reduce inhibited behavior, and increase the sense of social competence. And low level of SSE may hinder extensive interpersonal communications and constructive relationships with others. Existing literature shows that people who are confident in recognizing the needs of the victim and taking actions to meet these needs are more likely to help (Alessandri et al., 2009; Caprara et al., 2010). Consistently, Caprara (2002) has pointed out the crucial role of SSE in sustaining and promoting people’s prosocial engagement.

Previous studies indicate a positive association between agreeableness and SSE. Agreeableness is defined by several adjectives referring to cooperative, trusting, easy-going, soft-hearted, altruistic, and so on (Ashton et al., 1998; Liang and Chang, 2014). Highly agreeable individuals are willing to cooperate with others, tend to trust other people when contacting with them. Furthermore, highly agreeable individuals tend to perform better in controlling and regulating negative emotions, and dealing with interpersonal conflicts (Alessandri et al., 2009; Caprara et al., 2010). They display higher level of self-control and are prone to make constructive and cooperative responses in social situations (Caprara et al., 2012). These features may consequently lead the agreeable individuals to develop confidence in their capabilities to act prosocially. So, it is reasonable that agreeable people develop high level of SSE. Therefore we propose that SSE may mediate the agreeable-prosociality association.

### A Moderated Mediation Model

In addition to directly moderating the effect of agreeableness on PSB, previous literature further indicates that shyness may indirectly moderate such effect through reducing individuals’ SSE. That is, the relation between agreeableness and SSE can be moderated by shyness. Agreeable people tend to be more cooperative in social interactions, but this tendency can be reduced by shyness, a disposition associated with social anxiety and fear of negative evaluations. Shy individuals are more likely to use avoidant coping strategies because they have less chance to learn constructive ways of coping (Eisenberg et al., 1995b). They tend to avoid social interactions, and are less successful in activating or maintaining effective behavioral patterns in social settings. If these features are observed in an agreeable individual, he/she is less likely to develop self-confidence in social interactions (Wadman et al., 2008). This indicates that shyness can hinder people’s development of SSE, suggesting that the relation between agreeableness and SSE may be moderated by shyness.

Shyness and SSE are not two independent constructs. Previous literature suggests that shyness can be used as an antecedent of SSE. Thus, the moderation effect of shyness on the second stage of the mediation model (SSE → PSB) was not reasonable. In this study, only the “first stage” of the mediation model (agreeableness → SSE) was assumed to be moderated by shyness.

Based on these analyses, we established a moderated mediation model in which shyness moderated the pathway from agreeableness to SSE and PSB (PROCESS macro model 8 for SPSS; Hayes, 2012). We proposed that in non-shy relative to shy people, agreeable is more strongly associated with SSE, which is conductive to higher level of PSB. That is, the mediating effect of SSE in the agreeable-prosociality association is moderated by shyness.

### The Present Study

Have you ever experienced a situation that you saw someone in need, but you did not give a helping hand, even though you were willing to do so? If so, are you not an agreeable person? The answer may be “no.” Previous findings suggest that agreeableness may not necessarily exert positive effects on various types of PSB in different situations (Ben-Ner and Kramer, 2011; Oda et al., 2014). According to the social-cognitive perspectives (Hampson, 2012), a moderated mediation model was adopted in this study to infer the possible mechanisms by which agreeableness affects PSB, with the moderating role of shyness and the mediating role of SSE. Hampson (2012) proposed that a deep investigation of the underlying moderation and mediation mechanisms could lead to better understanding of how and why personality traits affect subsequent behavioral outcomes. In this study we are intended to infer the related mechanisms underlying the relation of agreeableness and PSB, which may provide theoretical explanations for inconsistent findings in previous studies.

It is noticeable that though the predictors in our moderated mediation model overlap in meaning to some extent, they have studied as different constructs (Cheek and Buss, 1981; Hill, 1989; Santesso et al., 2004; Caprara et al., 2012). For example, Caprara et al. (2012) found that agreeable people are more likely to develop greater confidence in their capacities in social engagement. Hill (1989) found that shy individuals tend to have lower level of SSE because they show greater concern of negative evaluations. More importantly, Cheek and Buss (1981) and Santesso et al. (2004), both have observed a sociability × shyness interaction in influencing behavioral outcomes, suggesting that the effects of personality traits can operate in an interactive manner rather in an additive manner.

In this study, a self-reported measure of PSB (the Self-Report Altruism Scale Distinguished by the Recipient; SRAS-DR; Oda et al., 2013) was used to assess PSB in daily life. Though observational and behavioral measures (e.g., helping in experimental situations, altruistic transfers in economic games) may be more objective, they only capture certain types of PSB in specific contexts and are not indicative of general prosocial engagement (Carlo and Randall, 2002; Burton-Chellew and West, 2013). For example, Benz and Meier (2008) found a weak correlation between experimental and field charitable behavior. SRAS-DR asks the participants whether they have act prosocially in daily life, classifying these PSB according to the recipients (i.e., family members, friends/acquaintances, and strangers). Given that the intention of this study is to assess prosociality in self-reported real social situations in view of the social characteristics of prosocial behavior, SRAS-DR may be a more appropriate tool which offers a better understanding of people’s general prosociality in social life.

**FIGURE 1:**
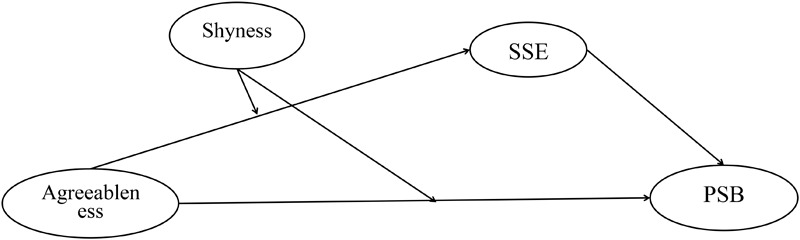
The hypothesized moderated-mediation model. PSB, prosocial behavior; SSE, social self-efficacy.

In this study we posit a moderated mediation model (see Figure 1). In this model, we assumed that shyness may moderate the relationship between agreeableness and PSB, such that agreeableness exerts a positive effect on PSB when the levels of shyness are low, but this effect become smaller when the levels of shyness are high (*Hypothesis* 1). We further assumed that shyness may indirectly moderate the association of agreeableness and PSB by reducing an individual’s SSE. That is, the relation between agreeableness and SSE may be moderated by shyness (*Hypothesis* 2). Specifically, shyness may diminish agreeable individuals’ SSE, thereby hindering their prosocial actions. Furthermore, because shyness is more strongly elicited in unfamiliar environments (Kagan, 2018), we assumed that shyness may have stronger effect when the recipients of PSB are strangers than that when the recipients of PSB are family or friends (*Hypothesis* 3*)*.

## Materials and Methods

### Participants and Procedure

Participants were 1383 undergraduate students (*N*_female_ = 817, *M*_age_ = 20.06, *SD*_age_ = 1.17) who were recruited via self-study courses from our university in 2018. The measures of agreeableness, PSB, shyness, and SSE (65 items in total) were administrated with the assistance of two trained research assistants. Participants were required to respond to all questionnaire items honestly according to their experience in daily life. The administration procedure lasted for approximately 25 min. The raw data can be found in Supplementary Material “Supplementary Data Sheet S1”. This study was conducted on the basis of the Helsinki declaration, with the ethical approval from the Institutional Review Board at Shandong Normal University. Written informed consent was obtained from all participants before measures. They agreed to participate in the current study and were told that they had the right to withdraw from the study at anytime.

### Measures

#### Agreeableness

Agreeableness is the most reliable predictor of a variety of PSBs in different situations, while other Big Five dimensions are only related to particular PSB (Habashi et al., 2016). Therefore in this study only the agreeableness sub-scale of the big five inventory-44 (BFI-44; Benet- Martínez and John, 1998) was used as the predictor. It contains nine 5-point Likert-format items (1 = disagree strongly, 5 = agree strongly) was used to assess agreeableness. The Chinese version of BFI-44 has been widely used and demonstrated acceptable psychometric properties (Srivastava et al., 2003; Guo et al., 2018a). An example item is “enjoy cooperating with others.” The total scores of all items were taken, with higher scores representing higher level of agreeableness. In this study, Cronbach’s alpha was 0.70.

#### Prosocial Behavior

The Chinese version of the Self-Report Altruism Scale Distinguished by the Recipient (SRAS-DR; Oda et al., 2013) was used to assess participants’ PSB. SRAS-DR contains 21 items designed to measure three types of PSB (toward family members, friends/acquaintances, and strangers), and each sub-scale consists of seven items. SRAS-DR evaluates a broad range of prosociality, including material help (e.g., lend money, carry luggage) and emotional support (listen to other’s complaints, accompany someone who is in bad mood). Participants were asked to indicate how frequently they engaged in such behaviors in daily life using a 5-point Likert-type scale (1 = never, 5 = very often). Example items are “I have helped my family members when they were not feeling well,” “I have helped my friends or acquaintances when they lost something,” and “I have offered help when a stranger was looking for something.” The Chinese version of SRAS-DR has acceptable psychometric properties (Feng and Guo, 2017). Sub-scales’ scores were taken, respectively, to represent the participants’ levels of PSB toward family members, friends, and strangers. In this study, Cronbach’s alpha of the whole scale and the abovementioned three sub-scales were 0.92, 0.87, 0.85, and 0.81, respectively.

#### Shyness

Shyness was measured by the Chinese College Students Shyness Scale (CCSSS; Wang et al., 2009) which was derived from the Henderson/Zimbardo Shyness Questionnaire (Henderson and Zimbardo, 2001). CCSSS contains 17 items each using a 5-point scale ranging from 1 (strongly unlike me) to 5 (strongly like me). An example of the items is “I blame myself when things do not go the way I want them to.” The total scores of all items were taken to represent participants’ level of shyness. Evidence shows that CCSSS has excellent psychometric properties (e.g., Guo et al., 2018b). In this study, Cronbach’s alpha was 0.91.

#### Social Self-Efficacy

Social self-efficacy was measured by the Scale of Perceived Social Self-efficacy (PSSE; Smith and Betz, 2000) which contains 18 5-point Likert-format items (1 = have no confidence, 5 = have confidence very much). An example of the items is “How much confidence do you have in making friends with your peers.” The Chinese version of PSSE also demonstrated satisfying psychometric properties (Meng et al., 2012). The total scores of all items were taken, with higher scores representing higher SSE. In this study, Cronbach’s alpha was 0.91.

#### Control Variables

Previous studies have demonstrated gender differences and age-related changes in PSB (Eisenberg et al., 1995a; Carlo and Randall, 2002). It was also suggested that family socioeconomic status significantly influenced people’s prosocial engagement (Benenson et al., 2007). Therefore, in this study, gender, age, and family socioeconomic status (i.e., parent’s education, per capita income) were regarded as control variables. Gender was entered as dummy variable by coding male as 1 and female as 0. The participants were also asked to report their father’s and mother’s education, respectively, using six options, namely, uneducated, primary school, middle school, high school, undergraduate, and postgraduate. Monthly household per capita income was rated using six options, namely, ¥500–999, ¥1000–1499, ¥1500–1999, ¥2000–2999, ¥3000–3999, and ≥¥4000.

## Results

Though common method variance (CMV) may not always be a grave concern in questionnaire survey (George and Pandey, 2017), procedural and statistical remedies (Chang et al., 2010) were still applied in this study. In the data collection stage, the participants were told that the results will be kept anonymously and confidentially. They were informed to complete the measures according to their true feelings and experiences, and were encouraged to respond honestly. Harman’s single-factor test (Podsakoff and Organ, 1986) was used to detect CMV because it is most widely used method and is sensitive under most conditions (Fuller et al., 2016). Exploratory factor analysis (EFA) of all 65 scale items yielded 12 factors with eigenvalue over one, and the first un-rotated component only explained 21.03% of the total variance, which is smaller than the criterion proposed by Podsakoff and Organ (1986). These suggested that the relationships among research variables in this study may not be contaminated by CMV.

### Descriptive Statistics and Correlation Analyses

Means, standard deviations, and Pearson correlations among research variables in this study were analyzed and presented in Table 1. Agreeableness was positively correlated with SSE and PSB toward family members, friends, as well as strangers, and negatively correlated with shyness. Shyness was negatively correlated with SSE, and PSB toward three types of recipients. SSE was positively correlated with PSB toward three types of recipients. In addition, parent’s educational level was not correlated with SSE and prosocial behavior. Therefore, we did not control this variable in moderated mediation analysis.

**Table 1:** Means, standard deviations, and correlations among research variables.

Variables	1	2	3	4	5	6	7	8	9	10
(1) Agreeableness	–									
(2) Shyness	-0.32**	–								
(3) Social self-efficacy	0.32**	-0.49**	–							
(4) PSB: family members	0.31**	-0.11**	0.38**	–						
(5) PSB: friends	0.31**	-0.08**	0.37**	0.78**	–					
(6) PSB: strangers	0.24**	-0.13**	0.36**	0.60**	0.53**	–				
(7) Gender	-0.11**	-0.01	0.01	-0.15**	-0.06**	-0.12**	–			
(8) Age	-0.02	0.05	-0.06*	0.03	-0.05	0.10**	-0.01	–		
(9) Per capita income	0.03	-0.10**	0.09**	0.02	0.05	0.02	0.07*	-0.04	–	
(10) Parent’s education	0.00	-0.05	0.02	-0.00	0.01	-0.03	-0.00	-0.15**	0.38**	-
*M*	33.33	52.66	60.49	29.72	30.12	25.81	0.41	20.06	3.52	7.17
*SD*	4.71	11.34	10.27	4.33	4.09	5.09	0.49	1.17	1.41	2.10

*^∗^*p* < 0.05, ^∗∗^*p* < 0.01; PC confidence level = 95%; PSB, prosocial behavior.*

### Moderation Effect of Shyness on the Relation of Agreeableness and PSB

**FIGURE 2:**
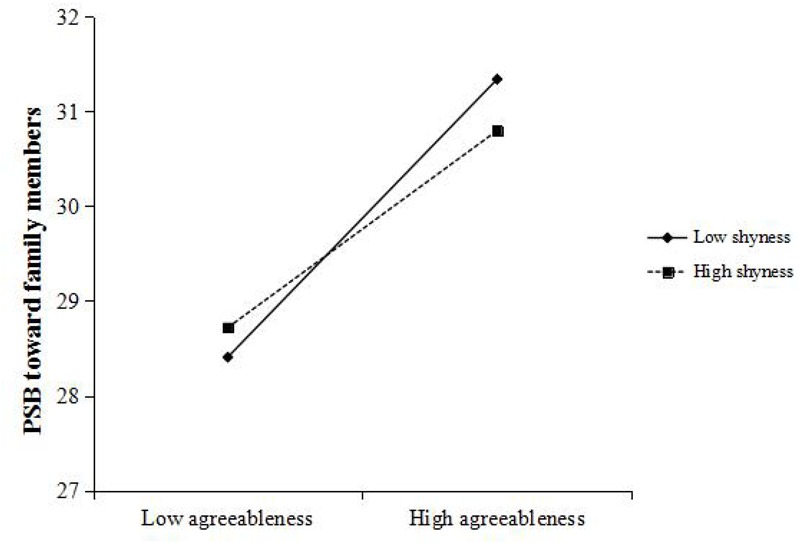
Relationship between agreeableness and PSB toward family members, moderated by shyness. PSB, prosocial behavior.

**FIGURE 3:**
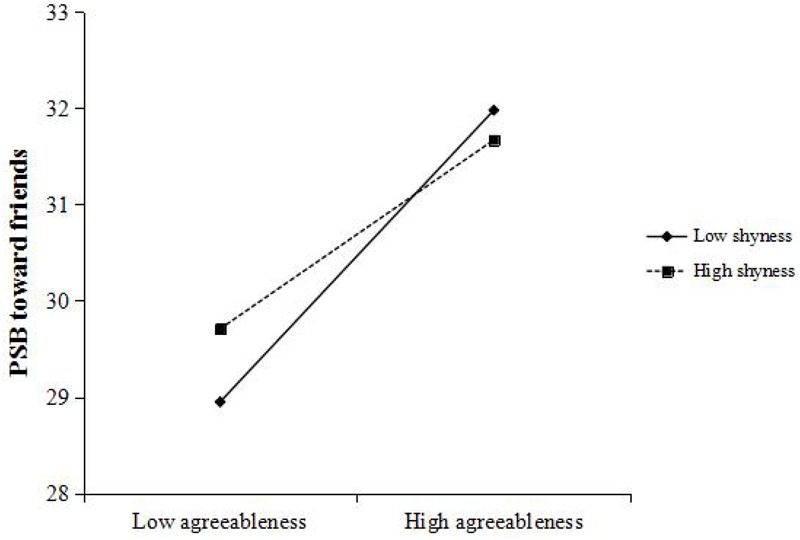
Relationship between agreeableness and PSB toward friends, moderated by shyness. PSB, prosocial behavior.

**FIGURE 4:**
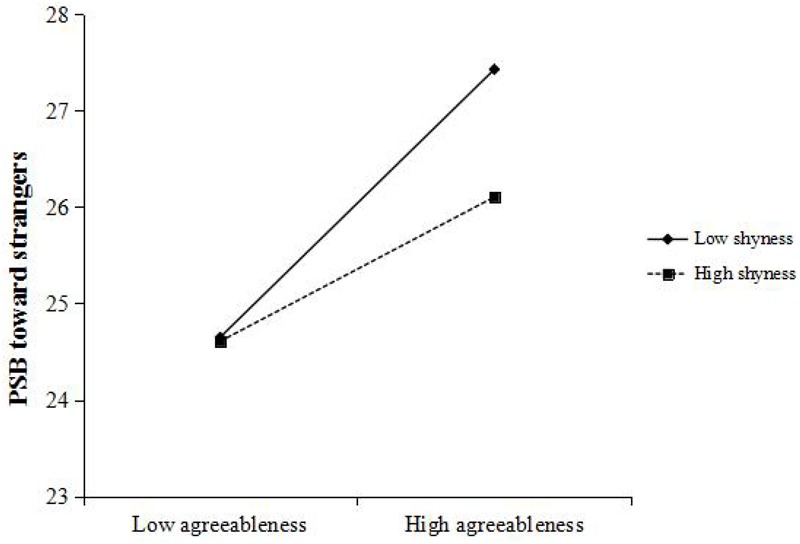
Relationship between agreeableness and PSB toward strangers, moderated by shyness. PSB, prosocial behavior.

Hayes (2012) PROCESS macro for SPSS was used to conduct moderation test. We applied Model 1 with 5,000 bootstrap samples in PROCESS macro to examine the moderation effect of shyness which will be indicated to be significant if 95% bootstrap confidence intervals of the agreeableness × shyness interaction do not include zero. Gender, age, and per capita income were entered as control variables. First, we examined the effects of agreeableness and shyness on PSB toward family members. The results indicated that the effect of agreeableness × shyness interaction on dependent variable was significant (*b* = -0.004, *t* = -2.270, *p* < 0.05, 95% CI = [-0.008, -0.001]). As is shown in Figure 2, the relationship between agreeableness and PSB toward family members was more positive under low level of shyness (*b* = 0.316, *t* = 9.518, *p* < 0.001, 95% CI = [0.251, 0.381]) than that under high level of shyness (*b* = 0.215, *t* = 6.218, *p* < 0.001, 95% CI = [0.147, 0.283]). Besides, the effect of gender on the dependent variable was significant, while the effects of age and per capita income were non-significant. Then, when the PSB toward friends was the dependent variable, the results showed that the effect of the interaction was significant (*b* = -0.005, *t* = -2.576, *p* < 0.05, 95% CI = [-0.009, -0.001]). As is shown in Figure 3, when the level of shyness was low, the relationship between agreeableness and PSB toward friends was significantly positive (*b* = 0.321, *t* = 9.665, *p* < 0.001, 95% CI = [0.256, 0.387]). While under high level of shyness, the effect of agreeableness on dependent variable was reduced to some extent but still significant (*b* = 0.209, *t* = 6.216, *p* < 0.001, 95% CI = [0.143, 0.275]). The effect of gender was also significant. Third, we tested the relationship between agreeableness and PSB toward strangers. The results indicated that the effect of the interaction was also significant (*b* = -0.006, *t* = -2.673, *p* < 0.01, 95% CI = [-0.010, -0.002]). Under low level of shyness (see Figure 4), agreeableness could significantly predict dependent variable (*b* = 0.293, *t* = 7.571, *p* < 0.001, 95% CI = [0.217, 0.369]). When shyness was high, though the effect of agreeableness on dependent variable was still significant, such effect was much weaker than that under low level of shyness (*b* = 0.159, *t* = 4.025, *p* < 0.001, 95% CI = [0.082, 0.237]). In addition, the effect of gender and age were significant in this analysis. These results supported *Hypothesis 1*. In order to examine whether shyness exerted stronger effect when the recipients of PSB are strangers, AMOS (version 20.0) was used to conduct multiple-group analysis. First, three models with the same structure (i.e., Path 1: agreeableness→PSB; Path 2: shyness→PSB; Path 3: agreeableness × shyness→PSB) were constructed according to three types of PSB. Then, a constrained model (three models had invariant structural weights on Path 3) and an unconstrained model were constructed. The result of model comparison indicated that there was no significant chi-square change between the constrained model and the unconstrained model (Δχ^2^ = 0.217, *p* > 0.05). This suggests that the effects of shyness on three types of PSB have no statistically significant difference. *Hypothesis* 3 was not supported.

### Moderated Mediation Analysis

Model 8 with 5,000 bootstrap samples in PROCESS macro was applied to conduct moderated mediation analysis. According to Muller et al. (2005), the moderated mediation model will be indicated if (a) agreeableness × shyness interaction could significantly predict PSB; (b) agreeableness × shyness interaction could significantly predict SSE; (c) SSE could significantly mediate the agreeableness-PSB association. The first criterion has been met. As for the second criterion, the result showed that agreeableness × shyness interaction exerted a significant effect on SSE (*b* = -0.008, *t* = -2.005, *p* < 0.05, 95% CI = [-0.016, -0.001]). The relation between agreeableness and SSE was more positive under low level of shyness (*b* = 0.489, *t* = 6.971, *p* < 0.001, 95% CI = [0.352, 0.627]) than that under high level of shyness (*b* = 0.307, *t* = 4.403, *p* < 0.001, 95% CI = [0.171, 0.444]). There was no significant effect of control variables on SSE. Table 2 shows the direct and indirect effects of agreeableness on PSB toward three recipients both in groups with low and high level of shyness. The results indicated that compared with low level of shyness, both direct and indirect effects of agreeableness on PSB were reduced when the level of shyness was high. These results support *Hypothesis* 2.

**Table 2:** Conditional direct and indirect effects of agreeableness on PSB.

Independent variable	Mediator	Dependent variable	Moderator (shyness)	Direct effect	95% bootstrap CI	Indirect effect	95% bootstrap CI
Agreeableness	Social self-efficacy	PSB toward family	High	0.166	[0.106, 0.227]	0.049	[0.027, 0.075]
			Low	0.238	[0.177, 0.299]	0.078	[0.054, 0.108]
		PSB toward friends	High	0.162	[0.106, 0.219]	0.047	[0.026, 0.070]
			Low	0.247	[0.189, 0.304]	0.075	[0.049, 0.100]
		PSB toward strangers	High	0.105	[0.032, 0.178]	0.054	[0.030, 0.083]
			Low	0.207	[0.133, 0.281]	0.086	[0.057, 0.121]

*PSB, prosocial behavior.*

**FIGURE 5:**
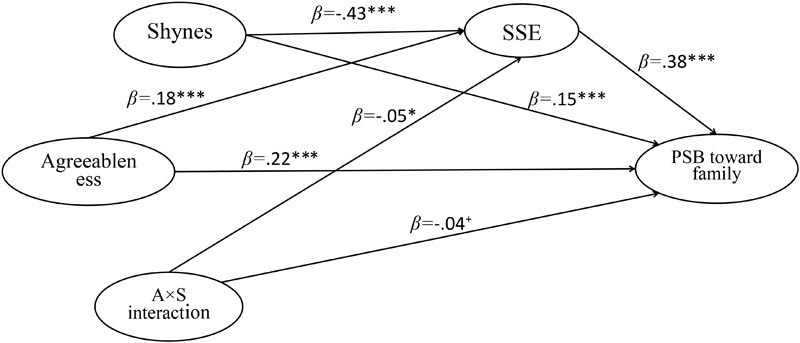
The standardized path coefficient of moderated mediation model (PSB toward family as dependent variable). 0.05 < ^+^*p* < 0.10; ^∗^*p* < 0.05, ^∗∗^*p* < 0.01, ^∗∗∗^*p* < 0.001; PSB, prosocial behavior; SSE, social self-efficacy; A × S interaction, agreeableness × shyness interaction.

**FIGURE 6:**
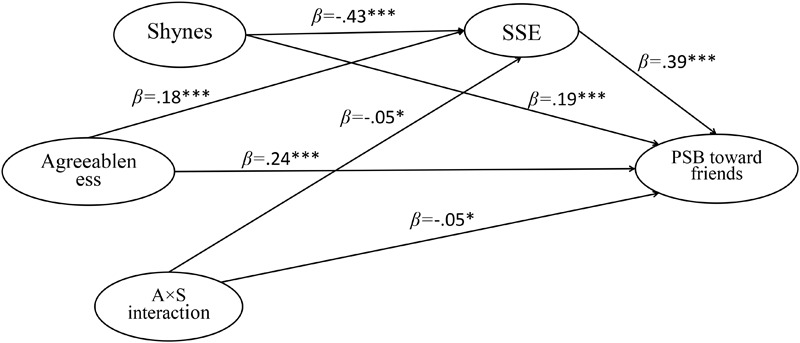
The standardized path coefficient of moderated mediation model (PSB toward friends as dependent variable). ^∗^*p* < 0.05, *p* < 0.01, ^∗∗∗^*p* < 0.001; PSB, prosocial behavior; SSE, social self-efficacy; A × S interaction, agreeableness × shyness interaction.

**FIGURE 7:**
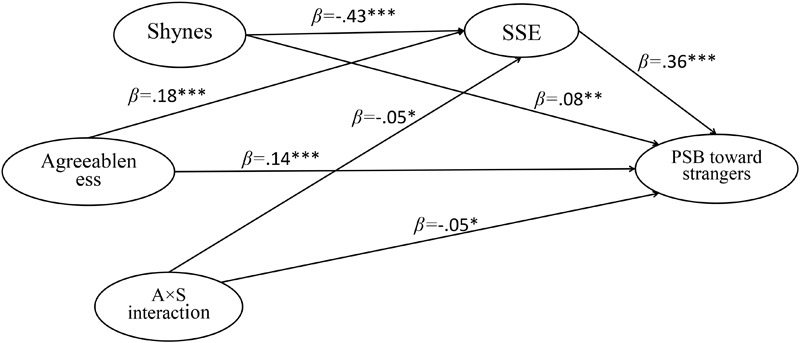
The standardized path coefficient of moderated mediation model (PSB toward strangers as dependent variable). ^∗^*p* < 0.05, ^∗∗^*p* < 0.01, ^∗∗∗^*p* < 0.001; PSB, prosocial behavior; SSE, social self-efficacy; A × S interaction, agreeableness × shyness interaction.

The AMOS software (version 20.0) allows us to illustrate the research results more intuitional and vividly. By constructing structural equation models (see Figure 5–7), we estimated the structural relations among research variables. The results indicated that SSE could significantly mediate the relation between agreeableness and PSB. And the agreeableness × shyness interaction could significantly predicted SSE (β = -0.05, *p* < 0.05) and three types of PSB (β = -0.04, 0.05 < *p* < 0.10; β = -0.05, *p* < 0.05; β = -0.05, *p* < 0.05).

## Discussion

Agreeableness has been considered as the core component of prosocial personality (Habashi et al., 2016). Agreeable people are cooperative, trusting, easy-going, soft-hearted, and altruistic (Ashton et al., 1998). They are prone to a positive perception of others, and tend to respond constructively in interpersonal interactions and sacrifice their interest to benefit others. Though the agreeableness-prosociality association has been confirmed by many studies, high agreeableness may not necessarily lead to prosocial engagement in all individuals (Caprara et al., 2012). In this study, we proposed that shyness may be the mechanism accounting for the different effect of agreeableness on PSB. In our moderated mediation model, we proposed that shyness may moderate the relationship between agreeableness and PSB, and moderate the mediation effect of SSE.

First, findings in this study confirmed the proposition that agreeableness may be core component of prosocial personality (e.g., Caprara et al., 2012; Habashi et al., 2016). The results showed a positive association between agreeableness and PSB toward all three types of recipients (i.e., family members, friends/acquaintances, and strangers). This is consistent with Ashton et al. (1998) who found that agreeableness could not only facilitate people’s kin altruism, but also promote their reciprocal altruism. But this is inconsistent with Oda et al. (2014) who found agreeableness only significantly predicted PSB toward friends/acquaintances.

Consistent with *Hypothesis* 1, we found that shyness moderated the relationship between agreeableness and PSB toward all three types of recipients. That is, an agreeableness by shyness interaction is related to less prosocial engagement (Bruch et al., 1999). As a positive social behavior, PSB always requires active interpersonal interactions. Shy individuals’ stronger tendency of avoiding disapproval can significantly hinder their prosocial engagement. This results were also be supported by previous studies. Twenge et al. (2007) argued that individuals with fewer friends were observed to possess less major prosocial skills. In addition, the finding that people who feel social exclusion were less likely to perform prosocial behaviors also provides evidence for our findings (Twenge et al., 2007). Therefore, even though some agreeable individuals have stronger prosocial emotions and motivations, high level of shyness may prevent them from taking actions. Consistent with this analysis, we observed the fact that the effect of agreeableness on PSB was significantly positive in non-shy people, but became smaller in shy ones, especially when the recipients are strangers. This indicates that whether and how agreeableness can translate into prosocial may partly dependent on an individual’s level of shyness. Specifically, agreeableness can be more strongly conductive to PSB in non-shy relative to shy individuals. This further confirmed the proposition that agreeableness alone cannot be responsible for all situational variability in PSB, especially when specific abilities are required (Caprara et al., 2012).

Given that the evolutionary explanations of PSB toward family, friends, and strangers are different (Ben-Ner and Kramer, 2011), such three types of PSB were regarded as three distinct constructs and have been estimated, respectively (Oda et al., 2013, 2014). For example, PSB toward family can be accounted for by kin selection (Hamilton, 1964), while PSB toward non-kin can be explained by reciprocity (Trivers, 1971). Because shyness is more strongly elicited in unfamiliar environments (Kagan, 2018), we supposed that the moderation effect of shyness may be stronger when the recipients of PSB are strangers. However, the result of multiple-group analysis did not support our Hypothesis. Although simple slope analysis revealed that the effect of shyness was stronger when recipients are strangers, and became smaller when recipients are family members and friends/acquaintances (see Figure 2–4), such differences did not reach statistical significance. Strong correlations among PSB toward family, friends, and strangers may explain the lack of differences in three moderation effects of shyness. Three types of PSB are different in nature (Ben-Ner and Kramer, 2011), but strong correlations among them (Table 1) indicate that prosociality is relatively stable across situations. In fact, a prior study has also drawn a consistent conclusion. For example, Oda et al. (2014) found that there also had robust correlations among three types of PSB.

Another goal of this study was to investigate the moderation effect of shyness on the mediation mechanisms. As have mentioned before, agreeable individuals’ prosocial engagement was influenced by their levels of shyness to some extent. Then, why are agreeable individuals high in shyness less likely to enact PSB than those with low level of shyness? To answer this question, we further tested the related mediation mechanisms to account for the moderating role of shyness. By introducing SSE as a mediator, we found that individuals with high level of shyness are less likely to develop high level of SSE, thereby inhibiting their prosocial engagement. Shy individuals often experience tension and anxiety, and are afraid of negative evaluation, which may weaken their self-confidence in interpersonal communication (Zimbardo, 1977; Kashdan and Roberts, 2004). PSB requires positive social interactions between the helper and the recipient. Therefore low SSE individuals are less likely to behave prosocially. Even though they are willing to help others, they may fear that their ineffective performance can put them in a negative light. On the contrary, people high in SSE tend to contact and communicate with a wide range of people. Thus, when they encounter someone asking for help, they are more likely to offer assistance (Bandura et al., 2003; Alessandri et al., 2009; Caprara et al., 2010; Guo et al., 2018a,b). These findings suggest that an agreeableness by shyness interaction is associated with greater social discomfort or lower SSE (Bruch et al., 1999), which is responsible for less PSB.

## Conclusions, Implications, Limitations, and Future Directions

This research may be the first study investigating the relationship between agreeableness and PSB by examining a moderated mediation model. The results showed that agreeableness could promote PSB, but this effect was influenced by shyness of an individual. Compared with agreeable but shy individuals, agreeable and non-shy individuals are more likely to turn their prosocial disposition into actual behaviors. In addition, this study confirmed the proposition that two traits may operate in an interactive manner in influencing social behavior (Cheek and Buss, 1981). Specifically, the interaction between agreeableness and shyness could lead to low level of SSE, which prevents shy individuals from behaving prosocially. That is, the agreeableness by shyness interaction could lead to less prosocial engagement via lower SSE.

Identifying the moderating role of shyness in the relation of prosocial personality and actual behavior may provide a foundation for encouraging more prosocial engagement. For shy individuals, some social situations are more anxiety provoking than other ones. Therefore we encourage them to engage in more social interactions with family members and close friends. These social engagements can facilitate the satisfaction of shy individuals’ psychological needs for autonomy, competence, and relatedness, and further enhance their SSE (Li et al., 2014). When shy individuals’ SSE is enhanced and social anxiety is reduced, they may act as prosocially as non-shy ones (Stoltenberg et al., 2013; Guo et al., 2018b). Engaging in PSB is especially beneficial for emotionally disturbed individuals (Alden and Trew, 2013), such as establishing desirable interpersonal relationships (Layous et al., 2012), reducing social avoidance goals and negative mood (Alden and Trew, 2013), enhancing social connections, and boosting well-being (Son and Wilson, 2012; Layous et al., 2017). Furthermore, enhanced well-being can attenuate social anxiety and inhibition in shy individuals (Li et al., 2014), leading to more positive social engagement. And this positive feedback loop can be continued.

The present study also has some limitations. First, participants in this study were Chinese undergraduates around 20 years old. Homogeneity of the study sample may prevent us from generalizing the findings to other populations. In future study, culturally diverse samples including Eastern and Western participants are expected to be involved in order to draw more sound conclusions. In addition, only self-reported measures were used to measure research variables. Alternative measures (e.g., observation, peer rating, field experiments, economic games) should be used in future studies. Lastly, this study adopted a correlational design using only cross-sectional data, which may yield biased parameter estimates for the moderated mediation model. Though the conceptual strength of such models has been tested, longitudinal or experimental data that have more power to detect causal relationships should be involved to test these models in future studies.

## EthicS Statement

This study was conducted on the basis of the Helsinki declaration, with the ethical approval from the Institutional Review Board at Shandong Normal University. Written informed consent was obtained from all participants before measures. They agreed to participate to the current study and were told that they had the right to withdraw from the study at anytime.

## Author Contributions

PS, ZL, and JF performed the survey. PS, QG, and ZL analyzed the data and contributed to the writing of the manuscript. ZL and JF contributed to the research materials and analysis tools. PS and QG contributed to the revised manuscript. QG made the final approval of the version to be published.

## Conflict of Interest Statement

The authors declare that the research was conducted in the absence of any commercial or financial relationships that could be construed as a potential conflict of interest.

## Supplementary Material

DATA SHEET S1 The raw data of this study which contains the research variables and demographic variables.
